# Therapeutic targets and functions of curcumol against COVID-19 and colon adenocarcinoma

**DOI:** 10.3389/fnut.2022.961697

**Published:** 2022-07-29

**Authors:** Jun Li, Peng Peng, Keng Po Lai

**Affiliations:** ^1^The Pharmaceutical Department, The Second Affiliated Hospital of Guangxi Medical University, Nanning, China; ^2^Department of Gastroenterology, The Second Affiliated Hospital of Guangxi Medical University, Nanning, China; ^3^Clinical Medicine Research Center, The Second Affiliated Hospital of Guangxi Medical University, Nanning, China

**Keywords:** colon adenocarcinoma, COVID-19, curcumol, bioinformatics, biological functions, pharmaceutics targets

## Abstract

Since 2019, the coronavirus disease (COVID-19) has caused 6,319,395 deaths worldwide. Although the COVID-19 vaccine is currently available, the latest variant of the virus, Omicron, spreads more easily than earlier strains, and its mortality rate is still high in patients with chronic diseases, especially cancer patients. So, identifying a novel compound for COVID-19 treatment could help reduce the lethal rate of the viral infection in patients with cancer. This study applied network pharmacology and systematic bioinformatics analysis to determine the possible use of curcumol for treating colon adenocarcinoma (COAD) in patients infected with COVID-19. Our results showed that COVID-19 and COAD in patients shared a cluster of genes commonly deregulated by curcumol. The clinical pathological analyses demonstrated that the expression of gamma-aminobutyric acid receptor subunit delta (GABRD) was associated with the patients' hazard ratio. More importantly, the high expression of GABRD was associated with poor survival rates and the late stages of COAD in patients. The network pharmacology result identified seven-core targets, including solute carrier family 6 member 3, gamma-aminobutyric acid receptor subunit pi, butyrylcholinesterase, cytochrome P450 3A4, 17-beta-hydroxysteroid dehydrogenase type 2, progesterone receptor, and GABRD of curcumol for treating patients with COVID-19 and COAD. The bioinformatic analysis further highlighted their importance in the biological processes and molecular functions in gland development, inflammation, retinol, and steroid metabolism. The findings of this study suggest that curcumol could be an alternative compound for treating patients with COVID-19 and COAD.

## Introduction

Since 2019, 537,591,764 confirmed coronavirus disease (COVID-19) cases, leading to 6,319,395 deaths worldwide, have been reported by WHO as of June 20, 2022 (https://covid19.who.int). Chronic health conditions were associated with the risk of COVID-19-related hospitalization and mortality (1). Patients with cancer are highly susceptible to severe symptoms (2). A systematic review of 52 pooled studies showed that patients with cancer and infected with COVID-19 exhibited a higher death risk (3). Similarly, Ahmadi's group showed that COVID-19 brings unfavorable survival outcomes for patients with colon cancer *via* the alteration of the immune cell infiltration-linked process (4), and the COVID-19 pandemic led to widespread disruption of colorectal cancer services (5). So, there is a need to identify alternative therapeutic compounds to reduce the severity and mortality rate of COVID-19 in patients with cancer. Colon adenocarcinoma (COAD), the most frequently diagnosed histological subtype of colorectal cancer, is one of the most prevalent malignant tumors in the gastrointestinal system worldwide (6, 7). The 5-year survival rate of patients with advanced COAD is <10% (8). Cumulating evidence has suggested the effectiveness of Traditional Chinese Medicines (TCMs) in cancer treatment (9, 10). One of the possible mechanisms is immune system regulation in patients with cancer (11). In addition, TCMs modulate the gut microbiota, which is considered a pathogenic factor of COAD (12). Besides the antitumor role, TCMs have also been reported to be effective in treating COVID-19 *via* their antiviral and anti-inflammatory activities (13, 14). Curcumol, a common TCM, is isolated from *Rhizoma curcumae*. The antitumor and antiviral effects of curcumol are well-documented (15, 16). Curcumol, in particular, has been shown to induce cell cycle arrest and increase the sensitivity of colon cancer to chemotherapy (17, 18). This study aimed to determine the pharmacological targets and the molecular mechanisms controlled by curcumol using network pharmacology and an *in vitro* COAD model. The results of this study will provide novel insight into the possible use of curcumol for treating patients with COAD and COVID-19.

## Materials and methods

### Identification of curcumol's targets against COAD and COVID-19

The transcriptome data from patients with COAD were obtained from The Cancer Genome Atlas (TCGA) database (https://portal.gdc.cancer.gov/) to identify COAD-associated genes. Using the DEseq2 package of R&Bioconductor, genes with a false discovery rate of <0.05 and a |logfold change| of >1 were considered differentially expressed genes (19). For the COVID-19-associated genes, keywords such as “coronavirus COVID-19,” “coronavirus disease 2019,” “severe acute respiratory syndrome coronavirus 2,” and “COVID-19” were subjected to databases search, including the Genecards Database, Online Mendelian Inheritance in Man Database (https://omim.org/), Therapeutic Target Database (20), Comparative Toxicogenomics Database (21), and National Center for Biotechnology Information (https://www.ncbi.nlm.nih.gov/). The pharmacological targets of curcumol were determined using various online tools and databases, including Swiss Target Prediction and Bioinformatics Analysis Tool for Molecular Mechanisms of Traditional Chinese Medicine (Batman-TCM) (22, 23). The target genes were subjected to UniProt for human database correction (24). The COVID-19-, COAD-, and curcumol-associated genes were compared and overlapped to obtain the potential curcumol's targets for treating COVID-19 and COAD.

### Clinicopathological analysis and functional characterization of curcumol's targets against COAD and COVID-19

To determine the pathological roles of the curcumol's targets in COAD, Cox proportional hazards models were applied in univariate survival analysis as a function of clinical variables and gene expression. The interaction of the identified curcumol's targets was analyzed using the STRING database (version 11.0) and Cytoscape (version 3.6.1) (25, 26). The functions and signaling pathways of curcumol's targets against COAD and COVID-19 were determined using Gene Ontology (GO) and Kyoto Encyclopedia of Genes and Genomes (KEGG) pathway enrichment analyses.

#### Cell culture study

The human lung adenocarcinoma cell line, A549, was incubated with high glucose Dulbecco's Modified Eagle Medium (DMEM, Solarbio, Beijing), 0.5% penicillin-streptomycin (Solarbio, Beijing), and 5% fetal bovine serum (Solarbio, Beijing) in 5% CO_2_ at 37°C.

#### Cell proliferation analysis

The cells were cultured in a 96-well plate at a cell density of 2 × 10^4^ cells/well and treated with different doses of curcumol (5, 25, and 75 μM) for 48 h. Following the co-incubation, cell proliferation was calculated using the cell-counting-kit-8 method (Beyotime Biotechnology, China), as reported previously (27).

#### Immunostaining procedures

After the curcumol treatment, A549 cells were fixed with a freshly prepared paraformaldehyde solution (4%, v/v) for 30 min at room temperature, followed by blocking with bovine serum albumin solution (5%, v/v) for 1 h at room temperature. The cells were then incubated at 4°C overnight with primary antibodies against BCHE or CYP3A4 (1:200, Bioss, Beijing, China). The secondary antibody with a fluorescent dye (Beyotime Biotechnology, China) was applied to bind the antigen–antibody complex. The cell nuclei were stained using 4', 6-diamidino-2-phenylindole dihydrochloride dye (Abcam, United States). The fluorescence-labeled positive cells were counted under the fluorescence microscropy system.

### Statistical analysis

The statistical data were expressed as the mean ± standard deviation. Comparisons between control and treatment groups were determined using the Statistical Product and Service Solutions (SPSS, 19.0 version) software (Chicago, IL, United States), followed by a one-way analysis of variance (ANOVA) using Tukey's *post-hoc* test. The statistical significance was identified as *p* < 0.05.

## Results

### Identification of pharmacological targets of curcumol for treating COVID-19 and COAD

**Figure 1:**
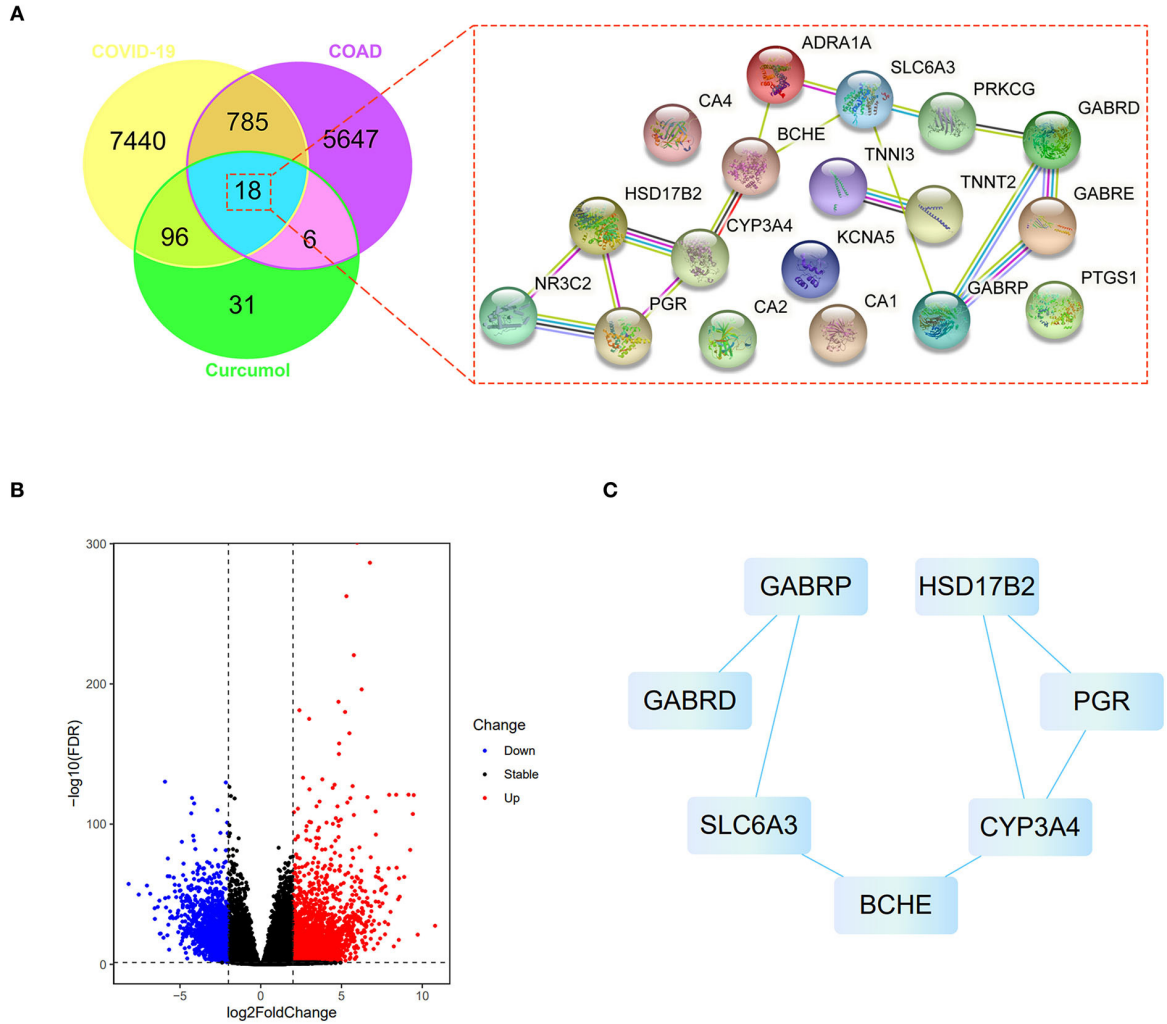
Network pharmacology identified the targets of curcumol against COVID-19 and colon adenocarcinoma. **(A)** Right panel: venn diagram shows the number of common targets of curcumol, COVID-19, and colon adenocarcinoma. Left panel: protein–protein interaction of curcumol's target against COVID-19 and colon adenocarcinoma. **(B)** Volcano plot shows the differentially expressed genes in colon adenocarcinoma targeted by curcumol. The blue dots represent the downregulated genes; the red dots represent the upregulated genes. **(C)** Cytoscape analysis highlights the protein–protein interaction of curcumol's core targets against COVID-19 and colon adenocarcinoma.

We searched the available online databases and identified 8,339 genes associated with COVID-19 (Figure 1A). To identify COAD-associated genes, we analyzed the transcriptome data of patients with COAD and obtained 6,456 differentially expressed genes (Figure 1A). When the COVID-19- and COAD-associated genes were compared, 803 shared genes were found (Figure 1B), of which 414 downregulated and 389 upregulated genes were identified in patients with COAD (Figure 1B). In addition, we identified 151 curcumol-associated genes from different databases (Figure 1A). Then, we compared the curcumol-associated genes with COVID-19/COAD-associated genes to determine the pharmacological targets of curcumol in COVID-19 and COAD. We found 18 target genes shared by curcumol, COVID-19, and COAD (Figure 1A). The molecular network analysis using Cytoscape further highlighted the protein–protein interaction of the 7 core targets, including solute carrier family 6 member 3 (SLC6A3), gamma-aminobutyric acid receptor subunit pi (GABRP), butyrylcholinesterase (BCHE), cytochrome P450 3A4 (CYP3A4), 17-beta-hydroxysteroid dehydrogenase type 2 (HSD17B2), progesterone receptor (PGR), and gamma-aminobutyric acid receptor subunit delta (GABRD) of curcumol against COVID-19 and COAD (Figure 1C; Supplementary Table 1).

### Clinicopathological analysis of curcumol's target genes for treating COVID-19 and COAD

**Figure 2:**
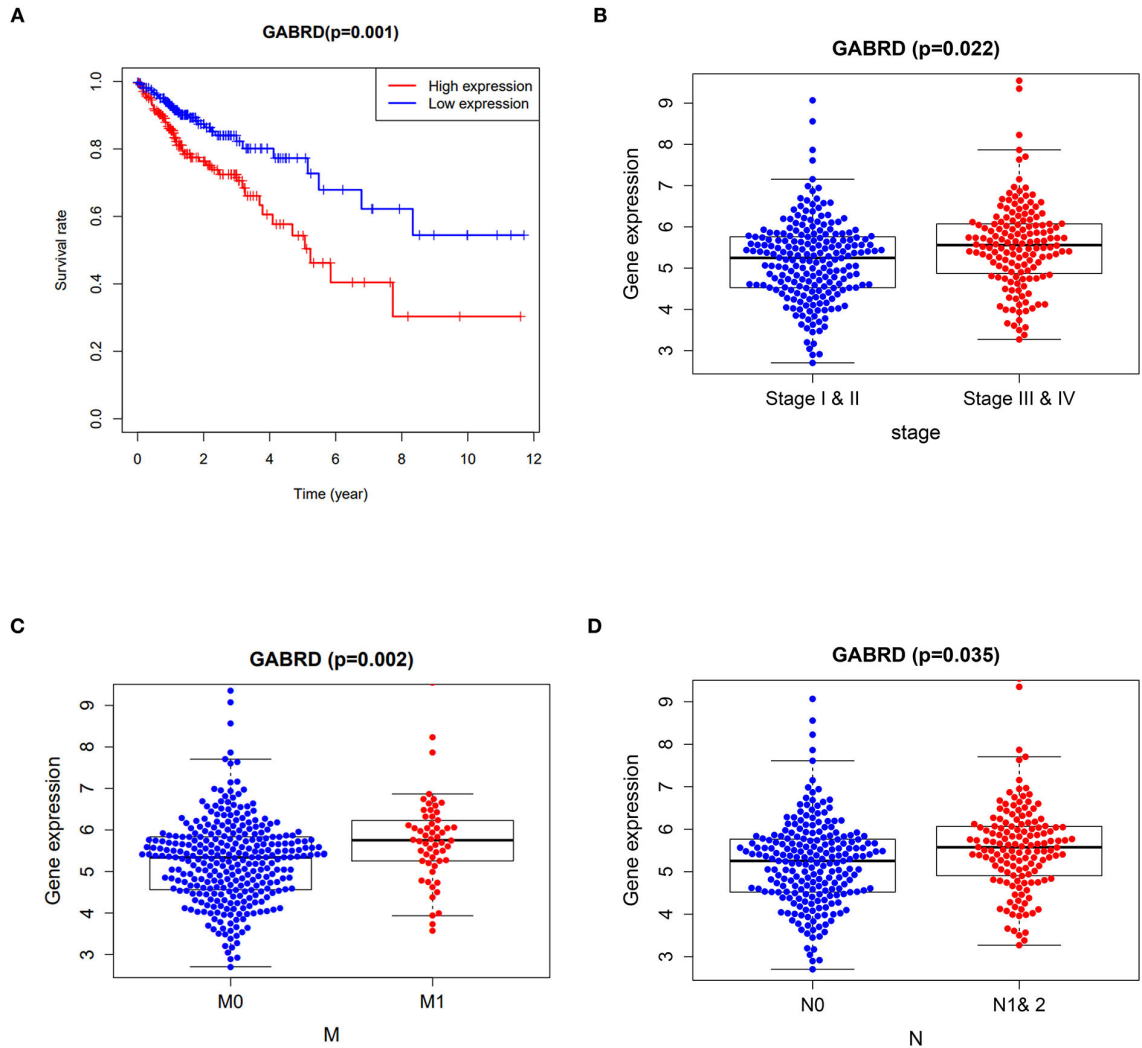
Clinicopathological analysis of curcumol's targets against COVID-19 and colon adenocarcinoma. **(A)** Survival analysis using the Kaplan–Meier estimator showed that the colon adenocarcinoma patients with higher expression of GABRD had poorer overall survival rates. Higher expressions of GABRD in colon adenocarcinoma patients are associated with **(B)** advanced stages, **(C)** metastatic tumors, and **(D)** a higher number of tumors spread to the lymph nodes. M0 means cancer has not spread to distant organs. M1 means cancer has spread to distant organs. N represents the number of lymph nodes containing the tumor.

Cox proportional hazards models were applied in univariate analysis of overall survival (OS) as a function of clinical variables and gene expression of the 7 core targets. Our results showed that the expression of GABRD was significantly associated with the hazard ratio of COAD (Supplementary Table 2). Survival analysis using the Kaplan–Meier estimator further highlighted that the COAD patients with higher expression of GABRD had a poorer OS rate (Figure 2A). In addition, the higher expression of GABRD was correlated with the later stage (Figure 2B), metastatic tumor (Figure 2C), and a higher number of tumors spread to the lymph nodes (Figure 2D) in COAD.

### Functional characterization of curcumol for treating COVID-19 and COAD

**Figure 3:**
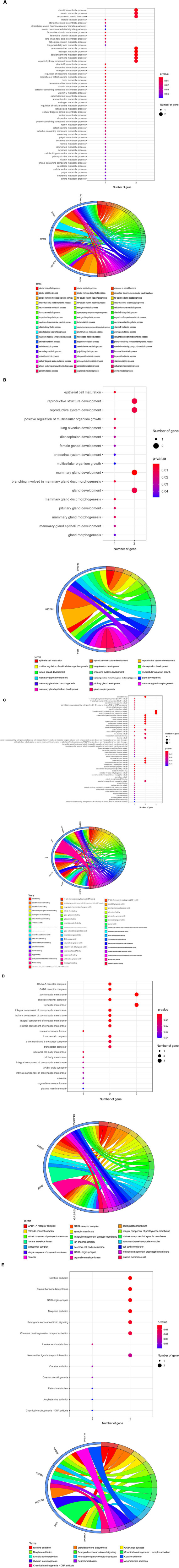
Functional characterization of curcumol's targets against COVID-19 and colon adenocarcinoma. The bubble plot highlights the involvement of curcumol's targets in biological processes related to **(A)** fat and steroid hormone biosynthesis, **(B)** gland development, and **(C)** steroid binding and ion channel activity. **(D)** Gene ontology showed the occurred cell components. **(E)** KEGG enrichment analysis showed the contribution of curcumol's targets in cell signaling pathways of carcinogenesis. The size of the bubble represents the number of genes. The color of the bubble represents the significance of the terms.

The core targets of curcumol were subjected to GO and KEGG enrichment analyses to determine their functional role in treating COVID-19 and COAD. In the GO analysis, our results highlighted the biological processes related to metabolism and biosynthesis, especially fat metabolism and biosynthesis, such as long-chain fatty acid biosynthetic and metabolic processes and fat-soluble vitamin metabolic processes (Figure 3A). In addition, steroid hormone biosynthesis and steroid hormone-mediated signaling pathways were found to be controlled by curcumol's targets (Figure 3A). More importantly, we found curcumol's targets' contributions to the biological processes related to growth and development, especially gland development (Figure 3B). In terms of molecular function, our results highlighted the steroid binding and activities such as steroid hormone receptor, steroid dehydrogenase, and steroid hydroxylase activities (Figure 3C).

Moreover, many molecular functions related to ion channels, such as ion channel, anion transmembrane transporter, sodium symporter, chloride symporter, and extracellular ligand-gated ion channel activities, were observed (Figure 3C). The observed biological processes and molecular functions occurred in the chloride channel complex, nuclear envelope lumen, ion channel complex, transmembrane transporter complex, transporter complex, organelle envelope lumen, and plasma membrane raft (Figure 3D). Finally, the KEGG pathway enrichment further highlighted the involvement of curcumol's targets in steroid hormone biosynthesis, retrograde endocannabinoid signaling, chemical carcinogenesis through receptor activation, and DNA adducts linoleic acid and retinol metabolism (Figure 3E).

### Curcumol treatment suppressed cell proliferation and altered the expression of BCHE and CYP3A4 in the COAD cell line

**Figure 4:**
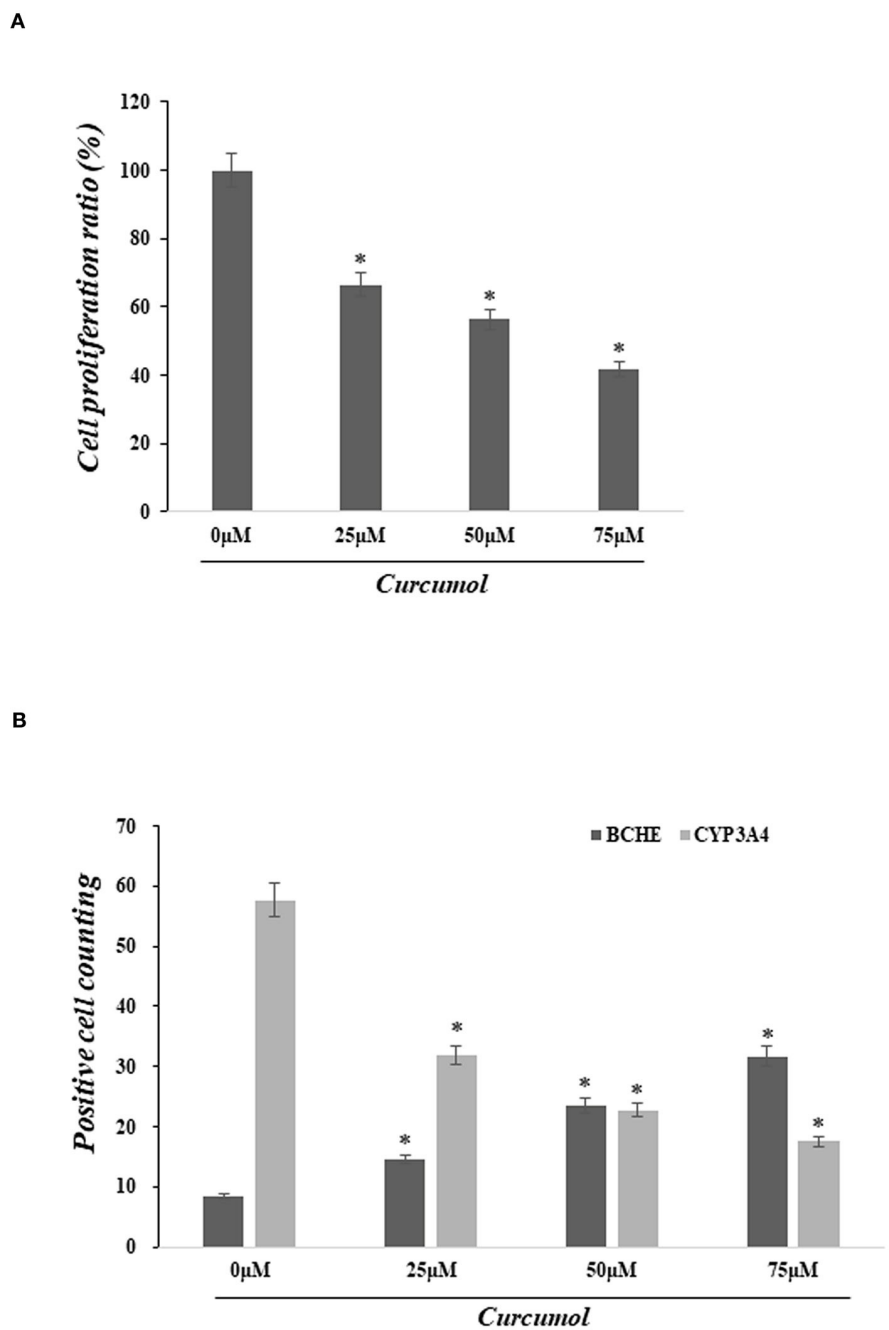
Curcumol treatment inhibited cell proliferation of colon adenocarcinoma and altered the expression of BCHE and CYP3A4. **(A)** MTT assay showed significant dose-dependent inhibition of cell proliferation in lung adenocarcinoma cell A594 caused by curcumol treatments (0–75 μM). **(B)** Immunofluorescence staining showed that the treatment of curcumol induced the expression of BCHE and reduced the expression of CYP3A4 in COAD cell, as compared to the control group. *p < 0.05.

Cell proliferation was determined using the MTT assay to assess the pharmacological action of curcumol on lung cancer cells, A549. Our data indicated that the treatments with curcumol caused a significant dose-dependent inhibition of cell proliferation in lung adenocarcinoma cells (Figure 4A). In addition, immunofluorescence staining analysis showed that curcumol treatment resulted in increased expression of BCHE and reduced expression of CYP3A4 in A549 cells, as compared to the control group (Figure 4B).

## Discussion

This study aimed to investigate the possible use of curcumol therapy for COVID-19 and COAD comorbidity. Using network pharmacology, we identified 7 core targets of curcumol against COVID-19 and COAD, including GABRD, GABRP, BCHE, CYP3A4, PGR, HSD17B2, and SLC6A3. Our clinicopathological analysis further suggested the prognostic value of GABRD in patients with COAD. GABRD, a subunit of GABAA receptor subtypes, has been reported to be associated with the development of many cancers (28). A clinical study suggested that GABRD promoted progression and predicted poor prognosis in colorectal cancer (29). In addition, gene set enrichment analysis further showed that the enhanced expression of GABRD predicted poor prognosis in patients with COAD (30). In our results, another GABA subunit, GABRP, was also found to be targeted by curcumol. It was reported that GABRP regulated macrophage recruitment and tumor progression in pancreatic cancer (31). In breast cancer, GABRP was found to control the stemness of triple-negative breast cancer cells through epidermal growth factor receptor signaling (32).

A study of the bronchial asthma mice model showed that the inhibition of GABRP could reduce the differentiation of airway epithelial progenitor cells into goblet cells, leading to reduced inflammation (33). So, the GABAA receptor subtypes GABRD and GABRP might be the promising targets of curcumol for treating COAD and COVID-19. BCHE, an α-glycoprotein synthesized in the liver, is abundant in the intestine and lung (34). The serum level of BCHE was reported to be decreased in many clinical conditions such as inflammation and infections (35). BCHE's low expression level has been documented in colorectal cancer (36), and its activities were found to be decreased in COAD patients (37). In addition, inhibition of BCHE is considered to reduce immunity through the cholinergic anti-inflammatory pathway, although its role in lung inflammation is still unknown (38). A rat study of sepsis suggested that BCHE can function as an inflammatory marker in sepsis (39), further supporting its role in inflammation. The other target of curcumol, CYP3A4, is a member of the cytochrome P450 superfamily of enzymes. Many clinical trials suggested the importance of the cytochrome P450 system in the drug discovery of COVID-19 (40–42) because CYP3A metabolism is altered in patients with COVID-19 having increased cytokine release. In addition, an *in vitro* study of colon cancer stem cells demonstrated the contribution of CYP3A4 in the chemoresistance of colon cancer and its negative impact on disease-free survival in the patients (43). So, targeting these genes with curcumol might provide an alternative approach for treating COVID-19 and COAD.

In the second part of our study, we focused on the functional roles of the curcumol targets. Our results highlighted the regulation of fat metabolism and biosynthesis by curcumol. Fat metabolism and excess visceral fat were reported to be closely associated with the severity of clinical outcomes in patients with COVID-19 (44, 45). It was further supported by an observational study and Mendelian randomization analysis that central fat distribution and metabolic consequences of excess weight are strongly associated with the severe COVID-19 outcomes (46). Furthermore, dietary fat and metabolism were reported to affect colonic tumorigenesis (47). A mice study showed that obesity is linked to altered metabolism in colon carcinogenesis through the JNK/STAT3-signaling pathway (48). Alex's group demonstrated that short-chain fatty acids stimulate tumor promoter angiopoietin-like 4 synthesis in human COAD cells (49).

## Conclusion

This study predicts the pharmacological targets of curcumol for treating COVID-19 and COAD. Additionally, the predicted targets involved in the biological and molecular functions related to fat metabolism and gland development are reported to be associated with the pathogenesis and severity of COVID-19 and COAD, suggesting the possible use of curcumol as a therapeutic compound for these diseases. The findings of this study need further validation by additional animal and preclinical studies before clinical use.

## Data availability statement

The original contributions presented in the study are included in the article/Supplementary material, further inquiries can be directed to the corresponding author.

## Author contributions

KL contributed to the conception, design of the manuscript, drafted this manuscript, and revised this manuscript. JL and PP contributed to the acquisition, analysis, and interpretation of data in this manuscript. All authors contributed to the article and approved the submitted version.

## Conflict of interest

The authors declare that the research was conducted in the absence of any commercial or financial relationships that could be construed as a potential conflict of interest.

## Publisher's note

All claims expressed in this article are solely those of the authors and do not necessarily represent those of their affiliated organizations, or those of the publisher, the editors and the reviewers. Any product that may be evaluated in this article, or claim that may be made by its manufacturer, is not guaranteed or endorsed by the publisher.

## Abbreviations

COAD: colon adenocarcinoma
COVID-19: coronavirus disease 2019
TCGA: The Cancer Genome Atlas
GO: gene ontology
KEGG: Kyoto Encyclopedia of Genes and Genomes
TCMSP: Traditional Chinese Medicine Systems Pharmacology Database and Analysis Platform
OS: overall survival
TCMs: Traditional Chinese Medicines
SLC6A3: solute carrier family 6 member 3
GABRP: gamma-aminobutyric acid receptor subunit pi
BCHE: butyrylcholinesterase
CYP3A4: cytochrome P450 3A4
HSD17B2: 17-beta-hydroxysteroid dehydrogenase type 2
PGR: progesterone receptor
GABRD: gamma-aminobutyric acid receptor subunit delta.
