# Supplementary material for: Therapeutic targets and functions of curcumol against COVID-19 and colon adenocarcinoma
Source: Front Nutr. 2022 Jul 29;9:961697. doi: 10.3389/fnut.2022.961697 (PMC9372556; doi:10.3389/fnut.2022.961697)
Supplement: Supplementary file 2 [file Table_2.DOCX]

Table2. Univariate Cox proportional hazards regression analysis of hubgene

| Gene Symbol | HR | HR.95L | HR.95H | p-value |
| --- | --- | --- | --- | --- |
| SLC6A3 | 1.006991 | 0.953674 | 1.063289 | 0.801814 |
| GABRP | 1.019921 | 0.938447 | 1.108469 | 0.642384 |
| BCHE | 1.025569 | 0.950492 | 1.106575 | 0.515103 |
| CYP3A4 | 0.972968 | 0.90077 | 1.050953 | 0.48604 |
| HSD17B2 | 0.956785 | 0.841268 | 1.088163 | 0.500989 |
| PGR | 1.045722 | 0.935255 | 1.169236 | 0.432533 |
| GABRD | 1.446563 | 1.171214 | 1.786646 | 0.00061 |
